# Long noncoding RNAs in head and neck cancer

**DOI:** 10.18632/oncotarget.12960

**Published:** 2016-10-27

**Authors:** Xiuhua Li, Yongbing Cao, Xiaojian Gong, Hongjiao Li

**Affiliations:** ^1^ School of Pharmacology, China Pharmaceutical University, Nanjing, Jiangsu, P. R. China; ^2^ Department of Stomatology,ChanghaiHospital, Second Military Medical University, Shanghai, P. R. China

**Keywords:** HNC, lncRNAs, function, mechanism, clinical application

## Abstract

Head and neck cancers (HNCs) include a series of malignant tumors arising in epithelial tissues, typically oral cancer, laryngeal cancer, nasopharynx cancer and thyroid cancer. HNCs are important contributors to cancer incidence and mortality, leading to approximately 225,100 new patients and 77,500 deaths in China every year. Determination of the mechanisms of HNC carcinogenesis and progression is an urgent priority in HNC treatment. Long noncoding RNAs (lncRNAs) are noncoding RNAs longer than 200 bps. lncRNAs have been reported to participate in a broad scope of biological processes, and lncRNA dysregulation leads to diverse human diseases, including cancer. In this review, we focus on lncRNAs that are dysregulated in HNCs, summarize the latest findings regarding the function and molecular mechanisms of lncRNAs in HNC carcinogenesis and progression, and discuss the clinical application of lncRNAs in HNC diagnosis, prognosis and therapy.

## INTRODUCTION

Head and neck cancer (HNC) is a class of malignant tumors that arise in the tissues of the upper neck, such as the tongue, gingiva, nasopharynx, larynx, and thyroid [1–3]. Head and neck squamous-cell carcinoma (HNSCC) constitutes the majority (nearly 90%) of HNCs, with smoking and alcohol consumption as the major risk factors. In recent years, accumulating evidence suggests that human papillomavirus infection is closely related to the occurrence of HNC [4]. HNC constitutes an important component of cancer incidence and mortality, leading to approximately 225,100 new patients and 77,500 deaths in China every year [5]. Although advanced therapeutics have been applied to treat HNC, the 5-five-year survival rate has not increased significantly and the mortality rate has not decreased significantly in recent decades [6]. HNC is usually discovered and diagnosed in the advanced stages, a factor that significantly contributes to the high mortality rates; however, current treatments may be ineffective in suppressing HNC progression and have many side effects. Early diagnosis and effective therapy play an important role in HNC treatment [7–9]. Elucidation of the mechanisms of HNC carcinogenesis and progression may enable the discovery of early diagnostic biomarkers and effective therapeutic targets.

Long noncoding RNAs (lncRNAs) are a focus of current research, particularly cancer research. lncRNAs are noncoding polyadenylated RNAs longer than 200 bps. The transcription of lncRNAs is typically executed by RNA polymerase II, and their expression is generally tissue-specific [10]. Previously, lncRNAs have been considered byproducts of a leaky transcriptional system, but recent findings suggest a different role. As shown in numerous reports, lncRNAs are indispensable and are involved in a spectrum of biological processes, including embryogenesis, stem-cell biology and cellular differentiation. Anomalous lncRNAs are suggested to be closely associated with multiple human diseases, including cancer [10–15]. For HNC, a distinct lncRNA expression profile between HNC tumors and normal human tissues has been identified and it evolves as the HNC progresses [16]. Research into the cellular functions of lncRNAs has verified that lncRNAs play significant roles in HNC carcinogenesis and that lncRNA-mediated biology is related to HNC progression. lncRNAs may serve as oncogenes or tumor suppressors by participating in the modulation of HNC cell proliferation, differentiation, migration and invasion [16, 17]. Some cancer-associated lncRNAs have well-characterized roles in HNC development, suggesting that lncRNAs can be used as new biomarkers for HNC diagnosis and prognosis and as therapeutic targets for HNC intervention [17].

In this review, we will focus on the lncRNAs that are dysregulated in HNC, including oral cancer, laryngeal cancer, nasopharynx cancer and thyroid cancer. Additionally, we will summarize the latest findings regarding the biological function and molecular mechanisms of lncRNAs in HNC carcinogenesis and progression and explore the clinical application of lncRNAs as targets for HNC diagnosis, prognosis and therapy.

## LNCRNAS IN HNC

Many lncRNAs are dysregulated in HNC and play important roles in HNC biology. Some lncRNAs that are up-regulated in HNC may act as oncogenes to promote cancer cell growth, migration, and invasion and to inhibit apoptosis. Some lncRNAs possess tumor-inhibiting functions and are down-regulated in HNC, accelerating oncogenesis and tumor development [16–18]. lncRNAs regulate gene transcription by associating with enzymes or protein complexes, thus participating in chromosome remodeling and epigenetic regulation. lncRNAs also regulate the transcription and translation of mRNAs by repressing miRNAs or altering mRNA modifications [10, 19, 20]. The biological functions and molecular mechanisms of specific lncRNAs in HNC are reviewed below (Table 1).

**Table 1:** Dysregulation of lncRNAs in HNC

lncRNA	Expression	Tumor type	Function and potential mechanism	Application
HOTAIR	up	LSCC, NPC, OSCC	Promotes invasion and inhibits apoptosis by inhibiting PTEN in LSCC [28]; promotes cell growth, migration and invasion in NPC by inducing VEGF-A and HSP70 expression [23, 32]; promotes proliferation, migration and invasion, and inhibits apoptosis by recruiting EZH2 to silence E-cadherin in OSCC [29, 30, 31].	diagnostic and prognostic biomarker [23, 28, 29, 91]
H19	up	NPC, HNSCC	Loss of imprinting in IGF2-H19 loci and abnormal H19 expression are associated with the oncogenesis and development of HNC [39–45]; H19 expression in HNC increases DNA methylation by repressing miR-148a-3p [46].	/
MEG3	down	TSCC	Inhibits cell proliferation and the cell cycle, promotes cell apoptosis; DNMT3B is the intermediary by which miR-26a regulates MEG3 expression in TSCC [51].	prognostic biomarker [51]
MALAT1	up	LSCC, NPC	Promotes cell proliferation and inhibits apoptosis in LSCC [63]; promotes cell proliferation, invasion, and metastasis in NPC; promotes cancer stem-cell by through acting as a ceRNA to regulate slug by reducing miR-1 activity in NPC [62].	/
UCA1	up	TSCC	Promotes migration in TSCC [65].	/
FOXCUT	up	OSCC	Promotes cell proliferation and migration in OSCC by regulating FOXC1 [67].	/
AFAP1-AS1	up	NPC	Promotes metastasis by increasing the levels of AFAP1 and some cytoskeleton-regulated proteins in NPC [73].	prognostic biomarker [73]
LET	down	NPC	Inhibits cell proliferation and promotes apoptosis; EZH2-mediated H3K27 methylation inhibits LET [77].	prognostic biomarker [77]
LOC401317	unknown	NPC	Inhibits tumor cell cycle progression by inducing P21 expression and repressing cyclin D1 and E1 expression; promotes cell apoptosis by inducing PARP and caspase 3 expression. lncRNA LOC401317 expression is induced by p53 [79].	/
PVT1	up	TC	Promotes cell proliferation and cell cycle progression in TC by recruiting EZH2 and regulating TSHR [80].	/
NAMA	down	PTC	Promotes cell apoptosis and arrests cell growth [82].	/
PTCSC2	down	PTC	Influences the expression of some genes involved in cancer and the cell cycle [83].	/
PTCSC3	down	PTC	Inhibits cell growth, influences the expression of genes involved in DNA replication, recombination and repair; cellular movement; tumor morphology and cell death [84].	/
NEAT1	up	LSCC	Promotes tumor growth and cell cycle progression in LSCC by regulating the miR-107/CDK6 pathway [71].	/
HNF1A-AS	up	NPC	Promotes cell proliferation, cell-cycle progression and migration by accelerating the EMT in NPC [75].	/
ROR	up	NPC	Promotes cell proliferation, migration and chemoresistance, and inhibits apoptosis by inhibiting the p53 signaling pathway [76].	/
GAS8-AS1	down	PTC	Suppresses tumor growth [78].	/
FAL1	up	PTC	Increases the risk of multifocality [87].	/
AB209630	down	HSCC	Promotes tumor growth and cell metastasis and invasion [88].	prognostic biomarker [88]
HOTTIP	up	TSCC	Unknown mechanism [85].	prognostic biomarker [85]
ENST00000438550	up	NPC	Unknown mechanism [86].	prognostic biomarker [86]
RP11-169D4.1-001	down	LSCC	Unknown mechanism [93].	prognostic biomarker [93]
AC026166.2-001	down	LSCC	Unknown mechanism [93].	prognostic biomarker [93]
LINC00312	down	NPC	Unknown mechanism [92].	diagnostic and prognostic biomarker [92]

Abbreviations: LSCC, laryngeal squamous-cell carcinoma; NPC, nasopharyngeal carcinoma; OSCC, oral squamous cell carcinoma; HNSCC, head and neck squamous cell carcinoma; TSCC, tongue squamous cell carcinoma; TC, thyroid cancer; PTC, papillary thyroid carcinoma; HSCC, hypopharyngeal squamous cell carcinoma.

### HOTAIR

HOTAIR (HOX antisense intergenic RNA) is a 2158-nucleotide mRNA-like lncRNA located at chromosome 12q13.13 that is transcribed from the antisense strand of the *HOXC* gene cluster by RNA polymerase II [21]. *HOTAIR* is not well conserved across species, and human *HOTAIR* contains 6 exons [22]. *HOTAIR* is up-regulated in many types of cancer and is related to oncogenesis, metastasis and poor prognosis in HNC, cervical cancer, and colon cancer [23–25]. HOTAIR recruits polycomb repressive complex 2 (PRC2) through its 5′-end binding domain and lysine-specific demethylase 1A (LSD1) through its 3′-end binding domain to targets to repress gene expression. The histone methylase enhancer of zeste homolog 2 (EZH2) is one component of PRC2, and the histone demethylase LSD1 is a flavin-dependent monoamine oxidase. HOTAIR interacts with EZH2 or LSD1, leading to gene silencing *via* H3K27-methylation or H3K4-demethylation, respectively [26, 27].

Recently, *HOTAIR* was shown to be dysregulated in HNC, including laryngeal squamous-cell carcinoma (LSCC), nasopharyngeal carcinoma (NPC) and oral squamous-cell carcinoma (OSCC) [28–32]. *HOTAIR* expression in LSCC tissues is 16-fold higher than in normal tissues, and increase in *HOTAIR* expression is statistically correlated with advanced tumor grade, lymph node metastasis (LNM), poor differentiation and advanced clinical stages. *HOTAIR* knockdown reduces cell invasion and increases cell apoptosis *in vitro* and inhibits LSCC xenograft growth *in vivo*. As revealed by a mechanistic study, *HOTAIR* serves as an oncogene by repressing phosphatase and tensin homolog deleted on chromosome ten (*PTEN*) expression to up-regulate DNA methylation. HOTAIR accumulation in cancer cells induces the hypermethylation of CpG islands in the *PTEN* gene, reducing the expression of *PTEN* at the mRNA and protein levels [28]. Because PTEN acts as a tumor repressor by inhibiting the phosphatidylinositol 3-kinase (PI3K) signaling pathway [33], the increased expression of *HOTAIR* in LSCC promotes cancer progression by activating the PI3K pathway [28]. *HOTAIR* expression is significantly increased in HNC and is correlated with cancer progression [29–32]. Additionally, in OSCC tumors, HOTAIR recruits EZH2 to the promoter region of E-cadherin and silences the expression of E-cadherin by increasing the levels of the H3K27me3 modification [30, 31]. HOTAIR is abundantly expressed in NPC tumor tissues and promotes tumor angiogenesis and growth. HOTAIR stimulates NPC angiogenesis by increasing the expression levels of the angiogenic factors vascular endothelial growth factor-A (VEGF-A) and glucose-regulated protein 78 (GRP78) [32]. GRP78, a member of the heat-shock protein 70 (HSP70) family, induces tumor angiogenesis and regulates the tumor microenvironment during tumor growth and metastasis [34, 35]. Furthermore, the expression of GRP78 in NPC increases the transcription of the angiogenic factors VEGF-A and angiopoietin 2 (Ang2) [32]. Thus, increased *HOTAIR* expression promotes cancer progression, and *HOTAIR* functions as an oncogene in HNC.

### H19

The discovery of H19 has attracted the attention of many scientists and prompted them to study lncRNAs. *H19* contains 5 exons and 4 introns and encodes a 2.3-kb noncoding RNA [11]. Insulin-like growth factor 2 (IGF2), a growth factor, stimulates tumor growth *via* autocrine or endocrine pathways [36]. Genomic imprinting is a type of hereditary epigenetic regulation that ensures the expression of parent-specific genes. Thus, only one of the parental imprinted alleles can be transcribed and translated [37]. *H19* and *IGF2* are a pair of imprinted genes on chromosome 11p15.5 that participate in embryonic development and growth regulation [11]. H19 gene is maternally expressed, and IGF2 is transcribed from the paternal allele. High *H19* expression has been observed during embryogenesis, but the transcripts are not expressed in most tissues after birth, with the exception of the heart and skeletal muscle [38]. However, loss of imprinting (LOI) and loss of heterozygosity (LOH) have been observed at the *IGF2-H19* locus in many cancers [39–45]. LOI at *H19* leads to the transcription of *H19* from the maternal and paternal alleles, and LOH at *H19* inhibits the expression of *H19*. Hypomethylation in the *H19* promoter region may be the main cause of *H19* LOI. Moreover, c-Myc also activates *H19* transcription in cancer by binding to the regulatory region in its gene. Mutation leads to *H19* LOH, and the expression of *H19* in cancer is down-regulated by p53 [40, 41]. Both LOI and LOH of *H19* are associated with cancer carcinogenesis and progression. Re-expression of *H19* in NPC suggests that *H19* might be an oncogene [39], and the down-regulation of *H19* in some HNSCC samples implies that H19 might be a tumor suppressor [42]. Although H19 has been enthusiastically studied for several years, the details of the mechanisms by which H19 functions as a tumor inhibitor or oncogene in cancer and its precise biological functions require further clarification.

Genomic imprinting plays important roles in organism development. LOI at the *IGF2-H19* locus and aberrant *H19* expression have been observed in HNC and may be related to HNC oncogenesis and progression [39, 42]. Among 64 HNSCC cases, 12 of 32 cases (37.5%) were showed a loss of imprinting at the *H19* gene and 11 of 27 cases (40.7%) showed a for loss of imprinting at the *IGF2* gene [44]. Among 27 samples from patients with Juvenile Nasopharyngeal Angiofibroma (JNA), 8 of 22 samples (36.4%) exhibited *H19* over-expression and 7 of 19 samples (36.8%) exhibited *IGF2* over-expression [43]. In another cohort of 35 HNSCC patients, 11 exhibited *H19* re-expression. Among patients showing *H19* expression, 6 had T2-grade tumors and 5 eventually presented recurrence and/or metastasis. In contrast, in patients without *H19* expression, including 5 with T2-grade tumors, only 1 exhibited regional recurrence [45]. Up-regulation of *H19* has been confirmed in undifferentiated NPC biopsies and LSCC tumor tissues and is related to low LSCC survival rates and cancer progression [39]. Hypomethylation of CpG dinucleotides in the *H19* promoter region may be the main epigenetic event that induces *H19* transcription [39]. The level of DNA methylation in the *H19* promoter region regulates *H19* expression, and the level of cellular DNA methylation is also regulated by H19. miR-148a-3p is a target of inhibition by H19, and miR-148a-3p reduces the levels of the DNA methyltransferase enzyme 1 (DNMT1) mRNA and protein, affecting cellular DNA methylation. Thus, H19 regulates the level of cellular DNA methylation through the H19/miR-148a-3p/DNMT1 cascade, and H19 may promote HNC cell migration, invasion and proliferation by increasing DNA methylation [46].

### MEG3

Maternally Expressed Gene 3 (MEG3), a tumor suppressor, is involved in the development and progression of many cancers [47–49]. *MEG3* is an imprinted gene located at chromosome 14q32 that encodes a 1.6-kb noncoding RNA from the maternal allele [50]. It is broadly distributed in many normal human tissues; however, MEG3 is not expressed in a series of human tumors and tumor cell lines, such as tongue squamous-cell carcinoma (TSCC), brain cancer, and hepatocellular cancer [47, 48, 51]. One of the main mechanisms of *MEG3* silencing is hypermethylation of the *MEG3* promoter region [52]. The loss of *MEG3* expression promotes tumor progression through specific molecular mechanisms, and re-expression of *MEG3* in tumor cells inhibits proliferation and induces apoptosis [51]. MEG3 inhibits tumorigenesis by increasing the expression of *P53* and Growth Differentiation Factor 15 (*GDF15*). p53 is a tumor suppressor protein, and GDF15 reduces tumor cell proliferation and tumor formation. MEG3 increases p53 levels by reducing its ubiquitin-proteasome-mediated degradation [53]. MEG3 can down-regulate the E3 ubiquitin ligase MDM2, which mediates p53 ubiquitination and increase its stability and expression levels in the cell. *GDF15* is also a target of MEG3, which can increase *GDF15* expression in a p53-dependent manner [53].

Low *MEG3* expression is associated with TSCC progression. MEG3 levels are significantly reduced in TSCC tissues compared with adjacent noncancerous tissues. Low MEG3 levels are associated with high TSCC mortality and poor overall survival. Over-expression of *MEG3* in SCC-15 and CAL27 cell lines inhibits cell proliferation, arrests the cell cycle and induces apoptosis [51]. The reduction of the miR-26a levels in TSCC down-regulates *MEG3* by increasing the DNA methylation levels. DNA methyltransferase 3B (DNMT3B) is a key enzyme in the DNA methylation process. *DNMT3B* mRNA contains a miR-26a target site in its 3′untranslated region (3′UTR), and miR-26a reduces the translation of *DNMT3B* by binding to its mRNA. Down-regulated of miR-26a in TSCC increases the level of DNMT3B, promoting DNA methylation and inhibiting *MEG3* transcription [51, 54].

### MALAT1

Metastasis-associated lung adenocarcinoma transcript 1 (MALAT1) was first identified in non-small cell lung cancer (NSCLC). Up-regulation of *MALAT1* in NSCLC is associated with cancer metastasis and is a biological marker used to predict disease outcomes in patients with early-stage NSCLC [55]. A MALAT1 transcript longer than 8,000 bps is abundantly expressed in the nucleus and is also known as nuclear-enriched abundant transcript 2 (NEAT2) [56]. MALAT1 was reported to be specifically up-regulated in many cancers, such as ovarian cancer, gastric cancer, and breast cancer, in several studies [57–59]. High *MALAT1* expression levels in cancer promote cell proliferation, migration and invasion. MALAT1 accelerates cancer progression by regulating the expression of some ‘metastatic signature’ genes [60]. MALAT1 regulates gene expression at the transcriptional level by affecting the alternative splicing process and modulating the location and phosphorylation states of serine/arginine (SR) splicing factors [61].

Higher *MALAT1* expression levels have been observed in LSCC tumor tissues and are significantly associated with a poor histological grade or an advanced clinical stage [30]. MALAT1 is also highly expressed in NPC tissues and it is closely related to LNM and advanced NPC stages [62]. Interference with *MALAT1* expression in cancer inhibits tumor growth and metastasis and increases tumor cell apoptosis and sensitivity to radiotherapy [62, 63]. The Slug protein plays a considerable role in elevating cancer stem-cell (CSC) activity and tumor radioresistance. MALAT1 increases the levels of the slug mRNA and protein by acting as a competing endogenous RNA. The binding of MALAT1 to miR-1 reduces the levels of miR-1 and the slug mRNA. MALAT1 eliminates miR1-induced slug gene silencing [64].

### Other lncRNAs in HNC

lncRNA urothelial cancer-associated 1 (UCA1) is involved in cancer invasion and metastasis. UCA1 is expressed at a dramatically higher level in TSCC tumor tissues than in neighboring non-tumor tissues, and patients with LNM express much higher levels of UCA1. High *UCA1* expression in TSCC increases cell migration [65]. The specific mechanisms by which UCA1 contributes to TSCC progression remain to be elucidated.

Forkhead box C1 (*FOXC1*) is a crucial cancer-related gene. FOXC1 promotes cancer progression by regulating the epithelial-mesenchymal transition (EMT), cell proliferation and migration [66]. FOXC1 regulates the expression of matrix metalloproteinases (MMPs) and VEGF-A. MMPs regulate cancer cell migration and VEGF-A promotes tumor angiogenesis. FOXC1 upstream transcript (FOXCUT) is a lncRNA that is substantially over-expressed in OSCC. Over-expression of *FOXCUT* in OSCC promotes tumor cell proliferation and migration by regulating *FOXC1* expression. The levels of the FOXC1 mRNA are positively associated with the levels of the FOXCUT transcript, and FOXCUT silencing substantially reduces *FOXC1* expression at both the mRNA and protein levels [67].

The lncRNA nuclear paraspeckle assembly transcript1 (*NEAT1*) is an oncogene in many malignancies, including LSCC [68–71]. High *NEAT1* expression is closely related to LSCC tumorigenesis, and patients with neck nodal metastasis exhibit high *NEAT1* expression. *NEAT1* knockdown in LSCC suppresses tumor growth, increases cell apoptosis and arrests the cell cycle. NEAT1 promotes LSCC tumorigenesis by regulating the miR-107/cyclin-dependent kinase 6 (CDK6) pathway [71]. The CDK6 protein belongs to the CDK family, regulating the transition from G1 to S phase of the cell cycle [72]. miR-107 represses CDK6 expression by targeting its 3′UTR. NEAT1 increases CDK6 expression by down-regulating miR-107 expression [71].

The lncRNA actin filament-associated protein 1 antisense RNA1 (AFAP1-AS1) is up-regulated in NPC and is related to NPC progression and poor patient survival. *AFAP1-AS1* knockdown represses NPC cell invasion and migration *in vitro* and inhibits NPC lung metastasis in nude mice. AFAP1-AS1 regulates AFAP1 and some cytoskeleton-regulated proteins, including proteins in the small GTPase-signaling Rho/Rac pathway [73]. AFAP1 regulates actin filament integrity and stimulates lamellipodia formation. Small GTPases modulate actin cytoskeleton formation, which is associated with cell shape and migration [74]. AFAP1-AS1 promotes NPC cell metastasis by affecting the integrity of actin filaments [73].

The lncRNA hepatocyte nuclear factor 1A-antisense RNA (HNF1A-AS) may modulate tumorigenesis in NPC. *HNF1A-AS* expression is much higher in NPC tumor tissues than in matched normal tissues. Over-expression of *HNF1A-AS* in NPC increases tumor cell proliferation, migration and cell cycle progression. HNF1A-AS may stimulate NPC tumor growth and metastasis by accelerating the EMT. In NPC, HNF1A-AS increases the levels of the mesenchymal proteins N-cadherin and vimentin and reduces the level of epithelial protein E-cadherin [75].

Radioresistance and chemoresistance are key drivers of cancer mortality, and the lncRNA-ROR is related to NPC chemoresistance. lncRNA-ROR expression is significantly increased in NPC tissues compared with in normal tissues. lncRNA-ROR expression in NPC cells is positively correlated with cell proliferation, metastasis and suppression of apoptosis. High lncRNA-ROR expression increase the ability of NPC cells to resist chemotherapy. lncRNA-ROR facilitates NPC chemoresistance and progression, possibly by inhibiting the p53 signaling pathway. lncRNA-ROR may constitute a medical target by which to decrease NPC chemoresistance [76].

lncRNA-LET (low expression in tumor) is a tumor suppressor that is down-regulated in NPC tumor tissue and NPC cell lines compared with paired non-cancer tissues and nasopharyngeal epithelial cells. LET down-regulation is significantly associated with an advanced NPC clinical stage, a greater tumor volume, an increased lymph node tumor burden, and a lower overall survival rate. Forced *LET* expression in CNE2 cells inhibits cell proliferation and increases cell apoptosis. Injection of LET-over-expressing cells into nude mice reduces tumor growth, and low lncRNA-LET levels are critically involved in NPC tumor cell proliferation. Further research suggests that low levels of LET expression are induced by EZH2-mediated H3K27 histone methylation in the LET promoter region [77].

A study of gene mutations in 402 Chinese NPC samples revealed that the growth arrest-specific 8-antisense RNA 1 (*GAS8-AS1*) is the second most commonly mutated gene and the lncRNA GAS8-AS1 may serve as a tumor inhibitor. The levels of the lncRNA GAS8-AS1 are significantly decreased in PTC. Increased *GAS8-AS1* expression substantially inhibits PTC cell viability. Furthermore, *GAS8-AS1* mutations in PTC are associated with an advanced clinical stage, and the wild-type lncRNA GAS8-AS1 is a stronger suppressor of tumor growth than mutated lncRNA GAS8-AS1 [78].

Recently, a novel lncRNA LOC401317 was shown to be up-regulated in human NPC cell lines over-expressing TP53, and the transcription of the lncRNA *LOC401317* is directly regulated by p53. As revealed in functional studies, LOC401317 is a tumor suppressor and LOC401317 transfection in NPC cells represses tumor cell proliferation, arrests the cell cycle and increases cell apoptosis. Mechanistic investigations revealed that LOC401317 inhibits the cell cycle in cancer cells by increasing the level of p21 and reducing the levels of cyclins D1 and E1. LOC401317 expedites apoptosis by inducing the activation of Poly (ADP-ribose) polymerase (PARP) and caspase-3 [79].

The levels of the lncRNA plasmacytoma variant translocation 1 (PVT1) are significantly increased in thyroid cancer (TC) tissues compared to neighboring normal tissues. *PVT1* silencing in TC cell lines obviously inhibits cell proliferation and arrests the cell cycle at the G0/G1 phase. PVT1 increases the mRNA and protein levels of thyroid-stimulating hormone receptor (TSHR) and the cell cycle-associated protein cyclin D1 [80]. Thyroid-stimulating hormone (TSH) and TSHR are associated with cell proliferation [81]. Furthermore, the lncRNA PVT1 recruits EZH2 to target genes, repressing the expression of some tumor suppressor genes. Recruitment of EZH2 and regulation of the TSHR levels may be the two mechanisms by which the lncRNA PVT1 facilitates TC oncogenesis [80].

A novel lncRNA, non-protein coding RNA, associated with MAP kinase pathway and growth arrest (NAMA), is, as its name suggests, related to the MAP kinase pathway and cell-growth arrest. NAMA levels are reduced in papillary thyroid carcinoma (PTC). *NAMA* over-expression in PTC cells promotes cell apoptosis and arrests the cell cycle. *NAMA* expression is induced by *BRAF* knockdown, suppression of the MAP kinase pathway, growth arrest and DNA damage. *NAMA* induction may be a molecular event or secondary signal that induces growth arrest and apoptosis [82]. However, further research is required to explore the functions and potential targets of NAMA.

Papillary thyroid cancer susceptibility candidate 2 (PTCSC2) and 3 (PTCSC3) are two novel thyroid-specific lncRNAs that may serve as tumor suppressors. The expression levels of *PTCSC2* and *PTCSC3* are strongly down-regulated in PTC tumor tissues and in TC tissues, respectively, compared with neighboring normal tissues. Over-expression of *PTCSC2* in PTC cells influences the expression of some cancer-related and cell-cycle-related genes. Re-expression of *PTCSC3* in TC cells represses tumor growth and influences the transcription of some genes associated with DNA replication, recombination, repair, cellular movement, tumor morphology, and cell death. Down-regulation of *PTCSC2* or *PTCSC3* is related to PTC or TC and increases the risk of developing PTC or TC. PTCSC2 and PTCSC3 may be involved in the genetic predisposition to PTC and TC. The polymorphisms rs965513 and rs944289 are related to PTC and TC risk *via* PTCSC2 and PTCSC3, respectively. The risk allele (A) of *rs965513* is significantly associated with down-regulation of *PTCSC2* in normal thyroid tissues. The risk allele (T) of *rs944289* is strongly related to the down-regulation of *PTCSC3* in TC tumor tissues [83, 84].

**Figure 1:**
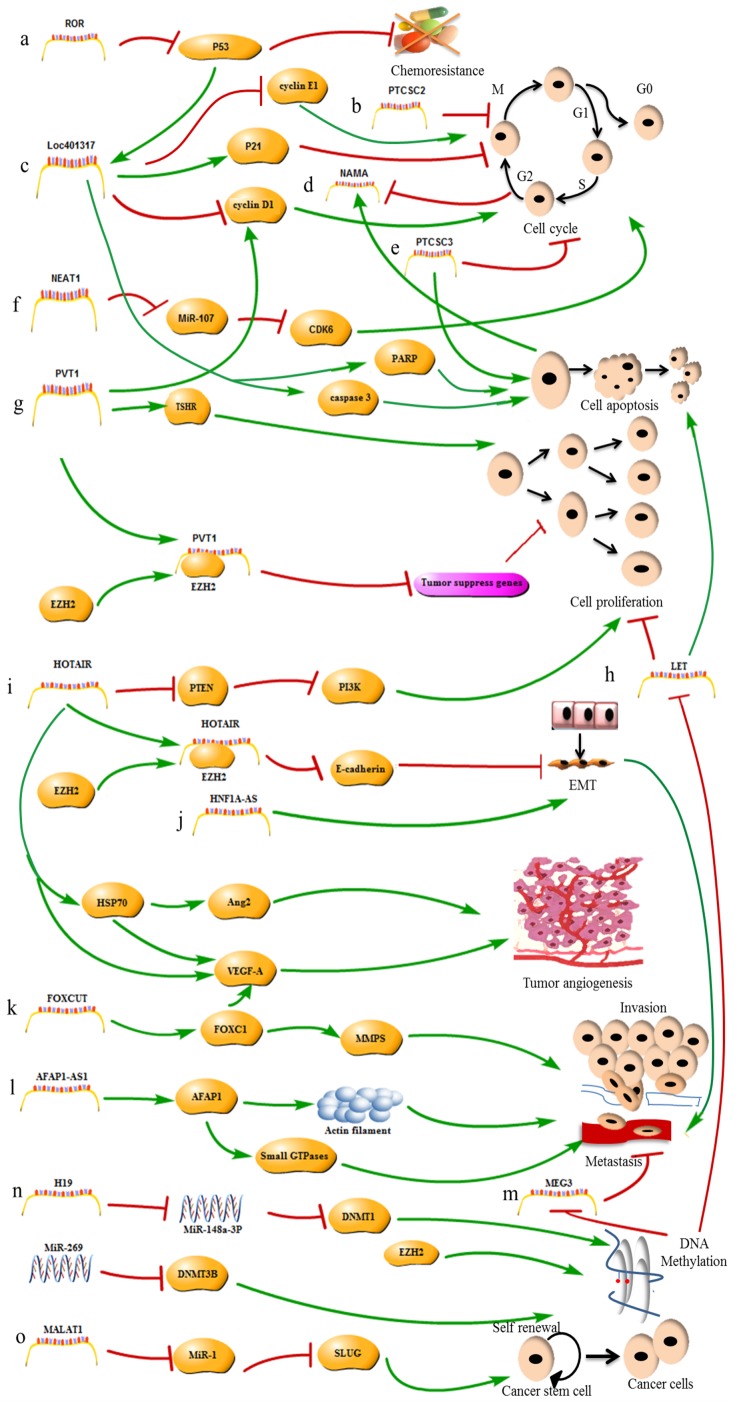
The mechanisms of lncRNAs in HNC **a**. The lncRNA-ROR promotes tumor chemoresistance by repressing P53 expression. **b**. The lncRNA PTCSC2 inhibits tumor cell cycle progression. **c**. The lncRNA LOC401317 inhibits tumor cell cycle progression by inducing P21 expression and repressing cyclin D1 and E1 expression, and promotes apoptosis by inducing PARP and caspase 3 expression; lncRNA LOC401317 expression is induced by p53 activity. **d**. The lncRNA NAMA represses tumor growth, and its expression is induced by cell cycle arrest and apoptosis. **e**. The lncRNA PTCSC2 inhibits tumor cell cycle progression and promotes apoptosis; **f**. The lncRNA NEAT1 promotes cell cycle progression by inhibiting the miR-107/ CDK6 pathway. **g**. The lncRNA PVT1 promotes tumor cell cycle progression by inducing cyclin D1 expression and promotes cell proliferation by inducing TSHR expression and recruiting EZH2 to repress the expression of tumor-suppressor genes. **h**. lncRNA-LET inhibits tumor cell proliferation and promotes apoptosis. LET is inhibited by EZH2-mediated H3K27 methylation. **i**. The lncRNA HOTAIR promotes cell proliferation by inducing PTEN expression, promotes metastasis by recruiting EZH2 to accelerate the EMT, and promotes tumor angiogenesis by inducing VEGF-A and HSP70 expression. **j**. The lncRNA HNF1A-AS promotes metastasis by accelerating the EMT. **k**. The lncRNA FOXCUT promotes invasion and metastasis by inducing FOXC1 expression. **l**. The lncRNA AFAP1-AS1 promotes metastasis by inducing AFAP1 expression. **m**. The lncRNA MEG3 promotes metastasis and is inhibited by miR-269-induced DNA methylation. **n**. The lncRNA H19 promotes tumorigenesis by regulating DNA methylation. **o**. The lncRNA MALAT1 increases CSC activity by inhibiting miR-1 expression.

The connection between the level of the lncRNA HOXA transcript at the distal tip (HOTTIP) and patients’ clinicopathological characteristics has been explored. High *HOTTIP* expression has been confirmed in TSCC and is positively correlated with high T classification, advanced clinical stages and distant metastasis [85]. The lncRNA ENST00000438550 was identified in an analysis of the differences in the lncRNA expression profiles between metastatic and primary NPC tumors. ENST00000438550 is strongly associated with the progression of NPC patients, and ENST00000438550 might be a prognostic biomarker and a potential treatment target in NPC [86]. An investigation of 100 PTC samples shows that focally amplified lncRNA on chromosome 1 (FAL1) is expressed at higher levels in PTC tissues than in surrounding healthy tissues. *FAL1* over-expression greatly increases the risk of multifocality, and FAL1 might be associated with PTC malignancy [87]. The lncRNA AB209630 may be a tumor suppressor in hypopharyngeal squamous cell carcinoma (HSCC). Down-regulation of *AB209630* promotes HSCC tumor growth, metastasis and invasion. HSCC patients with high AB209630 levels have a better prognosis than patients with low AB209630 levels. *AB209630* may be a valuable target in HSCC therapy [88]. lncRNAs play an important role in promoting HNC tumorigenesis and development. The known mechanisms by which some lncRNAs function in HNC are shown in Figure 1, and the as yet unknown mechanisms require further research.

## CLINICAL APPLICATIONS OF LNCRNAS IN HNC

lncRNAs are broadly involved in HNC oncogenesis and progression. The key roles of lncRNAs in HNC imply that lncRNAs may serve as biomarkers or therapeutic targets for HNC diagnosis, prognosis and treatment.

### Tumor diagnosis

The early diagnosis of HNC improves overall survival. lncRNAs play a vital role in the physiological and pathological progression of HNC, and the presence of lncRNAs in peripheral blood and body fluids suggests that they may be used as diagnostic biomarkers [89, 90].

### Diagnostic biomarkers in OSCC

Some well-documented lncRNAs, such as MALAT-1 and HOTAIR, are present in the saliva of OSCC patients. Patients with LNM exhibit higher *HOTAIR* expression in their saliva than patients with non-metastatic cancer. The presence of some lncRNAs in patients’ saliva may be associated with OSCC development, and measuring the lncRNA levels in saliva may provide a convenient method to rapidly and non-invasively diagnose OSCC [90]. *HOTAIR* expression in OSCC tissues is also correlated with a larger tumor size and advanced clinical stages, suggesting that HOTAIR may be used as a biomarker for diagnosing OSCC [29].

### Diagnostic biomarkers in LSCC

The combination of the serum levels of the HOTAIR and miR-21 exosomes is valuable for diagnosing LSCC. The serum levels of HOTAIR and miR-21 exosomes were higher in 52 LSCC patients than in 49 patients with vocal cord polyps, and high expression levels of miR-21 and HOTAIR in LSCC patients are significantly associated with advanced clinical stages and LNM. Moreover, the area under the receiver-operating characteristic (ROC) curve of the combination of HOTAIR and miR-21 is 87.6%, which is greater than the value observed for serum exosomes of miR-21 (80.1%) or HOTAIR (72.7%). The combination of HOTAIR and miR-21 might be a promising molecular marker to screen for LSCC [91].

### Diagnostic biomarkers in NPC

LINC00312 is distinctly down-regulated in NPC, and positive *LINC00312* expression is negatively correlated to the clinical classification and tumor size. *LINC00312* expression is used to distinguish healthy persons from NPC patients. Down-regulation of *LINC00312* may contribute to NPC oncogenesis and may be used to diagnose early-stage NPC. The dysregulation of lncRNAs in HNC may be a promising indicator for use in providing an exact and early cancer diagnosis [92].

### Tumor prognosis

The dysregulation of many lncRNAs is significantly related to HNC pathogenesis and prognosis. These molecules may be used as potential prognostic predictors, and their expression levels may predict patients’ disease outcomes [17].

### Prognostic biomarkers in OSCC

HOTTIP, MEG3, and HOTAIR could be used as independent prognostic parameters in TSCC. High *HOTTIP* expression has been observed in TSCC tissues, and patients with T3-4-grade tumors, distant metastasis or patients in clinical stages III-IV exhibit higher *HOTTIP* expression than patients with T1-2-grade tumors and no distant metastasis or patients in clinical stages I-II. *HOTTIP* over-expression is strongly associated with poor overall survival (OS) [85]. MEG3 expression is much lower in TSCC tumor tissues than in nearby nonmalignant tissues, and patients with low *MEG3* expression always have short survival times [51]. *HOTAIR* expression is higher in OSCC tumor tissue than in paired normal tissue, and high *HOTAIR* expression always indicates a relatively poor OS or disease-free survival (DFS) [29].

### Prognostic biomarkers in NPC

The lncRNAs HOTAIR, AFAP1-AS1 and ENST00000438550 could be used as prognostic biomarkers to predict disease outcomes in NPC patients [23, 73, 86]. Excessive AFAP1-AS1 levels have been detected in NPC samples compared with normal nasopharyngeal epithelium samples. *AFAP1-AS1* up-regulation is related to distant tumor metastasis and poor OS and relapse-free survival (RFS) [73]. *HOTAIR* expression is also higher in paraffin-embedded NPC biopsies than in non-tumor tissue samples, and high HOTAIR levels are associated with advanced clinical stages and a poor prognosis [23]. The *ENST00000438550* expression level is positively correlated with NPC progression, and high levels of ENST00000438550 are an indicator of NPC progression. Some down-regulated lncRNAs are also used as molecule tools to predict NPC outcomes [86]. The Kaplan-Meier method and log-rank test have been used to analyze the predictive potential of lncRNAs. Down-regulation of *LET* in NPC is related to poor RFS and OS, and low *LET* expression may predict a poor prognosis of NPC patients [77]. Positive *LINC00312* expression in NPC patients without LNM is significantly correlated with good DFS and OS; however, high *LINC00312* expression in patients with LNM is correlated with poor DFS and OS. LINC00312 may be a powerful independent indicator of NPC patient survival [92].

### Prognostic biomarkers in LSCC

The lncRNA HOTAIR might be a diagnostic marker for LSCC and an independent prognostic factor for LSCC [28]. HOTAIR levels are higher in LSCC tumor tissues than in adjacent non-neoplastic tissues and are significantly associated with shorter patient survival. HOTAIR has a major effect on LSCC progression and is an independent prognostic indicator [28]. Low expression levels of *AC026166.2-001* and *RP11-169D4.1-001* are statistically linked to OS in LSCC patients and are independent indicators of a poor prognosis [93]. Thus, lncRNAs may be used as prognostic factors to forecast cancer outcomes.

### Tumor treatment

The considerable importance of abnormal lncRNAs expression in HNC development and progression suggests that lncRNAs are potential therapeutic targets. Some tumorigenic lncRNAs are up-regulated in HNC and promote cancer growth. However, their cancer-promoting effects could be weakened by interference by siRNAs [28–31]. Reduction of the *HOTAIR* or *MALAT1* levels in LSCC inhibits cell proliferation and induces apoptosis [28, 63]. In addition, tumor-suppressing lncRNAs are expressed at low levels in cancer tissues, and transfection of an lncRNA expression vector in cancer cells to increase their expression inhibits tumor growth [50, 51]. *MEG3* expression is substantially reduced in OSCC tumor cell lines. Increased MEG3 levels in OSCC cells suppress cell proliferation, disrupt the cell cycle and stimulate apoptosis [51]. Thus, restoration of the expression of these lncRNAs in HNC may be a feasible therapeutic approach.

Some medical treatments for cancer target dysregulated lncRNAs and reduce the levels of aberrantly expressed lncRNAs in tumor tissues [93, 94]. The lncRNAs CDKN2B-AS1, HOTAIR and MALAT1 are up-regulated in LSCC. The levels of CDKN2B-AS1, HOTAIR and MALAT1 in LSCC cells are substantially reduced by cisplatin and paclitaxel in a concentration- and time-dependent manner [94]. lncRNA-MBL2-4:3 is significantly up-regulated and lncRNA-AL355149.1-1 is significantly down-regulated in TSCC tumor tissues. The expression levels of these lncRNAs are restored by the injection of 5-fluorouracil and paclitaxel in HN21B TSCC cells in a dose-dependent manner [93]. Thus, dysregulated lncRNAs are potential targets of HNC treatments.

lncRNAs are related to HNC radioresistance [95]. According to some analyses, the plasma levels of the lncRNA GAS5 in HNC patients respond to radical chemoradiotherapy. The pre- and post-radiotherapy GAS5 levels are significantly higher in HNC patients with a partial response to radiotherapy than in HNC patients exhibiting complete responses. Plasma GAS5 levels are a potential molecule biomarker for predicting radiotherapy responses in HNC patients [95]. *MALAT1* expression is substantially increased in NPC tissues. Increased *MALAT1* expression reduces NPC radiation sensitivity by improving the self-renewal of CSCs. *MALAT1* knockdown in NPC sensitizes the tumor cells to ionizing radiation and reduces the percentage of ALDH1-postive cells displaying properties associated with CSCs [64]. Differences in lncRNAs expression have been observed between NPC cells treated with ionizing radiation and NPC cells treated without ionizing radiation. Curcumin alters lncRNA expression in NPC cells while increasing the susceptibility of NPC cells to ionizing radiation [96]. lncRNAs play a significant role in cancer radioresistance. lncRNA-based therapeutics might represent a new direction for cancer treatment, although numerous experiments must be performed.

## CONCLUSIONS

Whole-genome sequencing studies have revealed that protein-coding genes constitute only a small portion, approximately 1.5%, of the genome. Most genes in the genome are transcribed into noncoding RNAs (ncRNAs), including lncRNAs, by RNA polymerase II [7]. An increasing number of reports indicate that lncRNAs significantly contribute to cancer biology, including HNC biology [15]. Dysregulated lncRNAs are significantly correlated with cancer development and progression in HNC [16]. The successful identification and characterization of lncRNAs may be a feasible strategy for studying lncRNAs in cancer. Microarray technology is one prospective method for screening irregular lncRNAs expression in malignant cancers. Knockdown or over-expression of certain lncRNAs *in vitro* and *in vivo* may reveal their functions and mechanisms in cancer biology. Some lncRNAs modulate gene expression at the transcriptional or post-transcriptional levels [61]. Some lncRNAs interact with DNA, RNA or proteins to exert their gene-regulating functions and affect the behavior of cancer cells. However, lncRNAs have multiple intricate functions and molecular mechanisms, and only a small fraction of lncRNAs and their functions in cancer biology have been discovered and investigated. In addition, the functions of the few known lncRNAs remain to be investigated in detail and additional functional lncRNAs remain to be identified.

Differential lncRNA expression between HNC tumors and normal tissues suggests that lncRNAs have potential for use as sensitive biomarkers in HNC detection and prognosis [16]. For example, high HOTAIR expression levels are related to HNC development, and lower levels of EMG3 are related to cancer metastasis [31, 51]. The correlations between lncRNA expression in patient saliva and the clinical stages of OSCC provide markers for a noninvasive and rapid diagnosis [90]. Over-expression of some tumor-promoting lncRNAs and reduced expression of some tumor-suppressing lncRNAs are related to poor OS and DFS. Cancer-related lncRNAs could be used as independent prognostic parameters in HNC [51, 91]. Although some reports have identified lncRNAs in body fluids [90, 91], the stability of peripheral lncRNAs remains largely unknown and lncRNA degradation may restrict the use of lncRNAs as biomarkers. Although some lncRNAs are negatively or positively correlated with the outcomes of HNC patients, the analysis of large samples is required to assess the utility of lncRNAs in clinical predictions.

As reported in numerous studies, dysregulation of lncRNAs plays a major role in HNC development, and lncRNAs are promising novel therapeutic targets for cancer intervention [4] because oncogenic lncRNAs are up-regulated and tumor-suppressing lncRNAs are down-regulated in HNC. Thus, the modulation of ectopic lncRNA expression is one direction for the development of lncRNA-based cancer therapies. For example, knockdown of *HOTAIR* or *MALAT1* with siRNAs inhibits cancer-cell proliferation and increases apoptosis [28, 63]. Although success has been achieved in cell-based experiments in which lncRNAs served as therapeutic targets, lncRNA-targeted cancer therapies must overcome many obstacles before its implementation, and a tremendous amount of research is required to elucidate lncRNA structures, folding, functions, mechanisms and interactions with other molecules. The development of lncRNA-targeted therapies will require a detailed knowledge of the functions of lncRNAs in HNC.

In summary, dysregulated lncRNAs play an essential role in HNC tumorigenesis and progression. They may serve as oncogenes or tumor inhibitors, and further research is needed to determine their precise, detailed mechanisms and functions. The importance of lncRNAs in cancer suggests the possibility of developing lncRNA-oriented clinical applications. Although this problem presents some difficulties, there is also the strong possibility of significant advancements in the near future.

## Abbreviations

HNC: head and neck cancer
HNSCC: head and neck squamous cell carcinoma
lncRNAs: long noncoding RNAs
HOTAIR: HOX antisense intergenic RNA
PRC2: polycomb repressive complex 2
LSD1: lysine-specific demethylase 1A
EZH2: histone methylase enhancer of Zeste homolog 2
LSCC: laryngeal squamous cell carcinoma
NPC: nasopharyngeal carcinoma
OSCC: oral squamous cell carcinoma
LNM: lymph node metastasis
PTEN: tensin homolog deleted on chromosome ten
PI3K: phosphatidylinositol 3-kinase
VEGF-A: vascular endothelial growth factor-A
GRP78: glucose-regulated protein 78
HSP70: heat shock protein 70
IGF2: insulin-like growth factor 2
LOI: loss of imprinting
LOH: loss of heterozygosity
DNMT1: DNA methyltransferase enzyme 1
TSCC: tongue squamous cell carcinoma
GDF15: growth differentiation factor 15
DNMT3B: DNA methyltransferase 3B
MALAT1: metastasis-associated lung adenocarcinoma transcript 1
NSCLC: non-small cell lung cancer
NEAT2: nuclear-enriched abundant transcript 2
SR: serine/arginine
CSC: cancer stem cells
UCA1: urothelial cancer-associated 1
FOXC1: forkhead box C1
FOXCUT: FOXC1 upstream transcript
EMT: epithelial-mesenchymal transition
NEAT1: nuclear paraspeckle assembly transcript1
AFAP1-AS1: actin filament associated protein 1-antisense RNA1
HNF1A-AS: hepatocyte nuclear factor 1A-antisense RNA
GAS8-AS1: growth arrest-specific 8-antisense RNA 1
PARP: poly (ADP-ribose) polymerase
PVT1: plasmacytoma variant translocation 1
TSHR: thyroid-stimulating hormone receptor
TSH: thyroid-stimulating hormone
NAMA: non-protein coding RNA, associated with MAP kinase pathway and growth arrest
TC: thyroid cancer
PTC: papillary thyroid carcinoma
PTCSC2: papillary thyroid cancer susceptibility candidate 2
PTCSC3: papillary thyroid cancer susceptibility candidate 3
HOTTIP: HOXA transcript at the distal tip
FAL1: focally amplified lncRNA on chromosome 1
HSCC: hypopharyngeal squamous cell carcinoma
ROC: receiver-operating characteristic
OS: overall survival
DFS: disease-free survival
RFS: relapse-free survival
ncRNAs: noncoding RNAs
